# Tetrahydrocannabinol-Rich Extracts From *Cannabis Sativa* L. Improve Glucose Consumption and Modulate Metabolic Complications Linked to Neurodegenerative Diseases in Isolated Rat Brains

**DOI:** 10.3389/fphar.2020.592981

**Published:** 2020-11-24

**Authors:** Ochuko L. Erukainure, Motlalepula G. Matsabisa, Veronica F. Salau, Md. Shahidul Islam

**Affiliations:** ^1^ Department of Pharmacology, School of Clinical Medicine, Faculty of Health Sciences, University of the Free State, Bloemfontein, South Africa; ^2^ Department of Biochemistry, School of Life Sciences, University of KwaZulu-Natal, Durban, South Africa

**Keywords:** brain glucose consumption, *Cannabis sativa*, neurodegenerative diseases, tetrahydrocannabinol 3, carbohydrate metabolism

## Abstract

Reduced brain glucose consumption arising from impaired glucose uptake and utilization has been linked to the pathogenesis and complications of neurodegenerative diseases. The ability of *Cannabis sativa* L. tetrahydrocannabinol (THC)-rich extracts to stimulate brain glucose uptake and utilization as well as its modulatory effect on gluconeogenesis, antioxidative, purinergic and cholinergic activities were investigated in isolated rats’ brains. *C. sativa* leaves were sequentially extracted to yield the hexane and dichloromethane extracts. The extracts were incubated at 37°C with freshly harvested brains in the presence of glucose for 2 h. The control consisted of incubation without the extracts, while brains without the extracts and glucose served as the normal control. Metformin was used as the standard drug. *C. sativa* extracts caused a significant (*p* < 0.05) increase in brain glucose uptake, with concomitant elevation of glutathione level, superoxide dismutase, catalase, and ecto-nucleoside triphosphate diphosphohydrolase activities compared to the controls. Incubation with *C. sativa* extracts also led to depletion in malondialdehyde and nitric oxide levels, acetylcholinesterase, butyrylcholinesterase, glucose 6-phosphatase and fructose-1,6-biphosphatase activities. GC-MS analysis of the extracts revealed the presence of THC. *In silico* analysis predicted THC to be permeable across the blood-brain-barrier. THC was also predicted to have an oral LD_50_ and toxicity class values of 482 mg/kg and 4 respectively. These results indicate that *C. sativa* improves glucose consumption with concomitant suppression of oxidative stress and cholinergic dysfunction, and modulation of purinergic and gluconeogenic activities in brain tissues

## Introduction

The brain’s dependence on glucose for energy generation is well documented. Brain glucose homeostasis has also been reported to be important for neuronal generation and maintenance, regulation of neurotransmitter, cognitive function and synaptic plasticity (Neumann et al., 2008). Glucose transporters aid in transporting glucose across the blood brain barrier (BBB) from the blood stream to the brain. Altered glucose homeostasis in the central nervous system (CNS) has been reported in most neurodegenerative diseases such as Alzheimer’s and Parkinson diseases (An et al., 2018). This has been attributed to abnormalities in insulin signaling pathways in the brain as well as alteration of the glucose transporters at the BBB (Gejl et al., 2017; An et al., 2018). These abnormalities and alterations often cause deceased brain glucose consumption which can lead to a hypometabolic brain state characterized by glucose dysmetabolism (Zilberter and Zilberter, 2017). Thus, making the brain susceptible to degenerative diseases. This is evident in studies which correlated the risk of Alzheimer’s disease with reduced brain glucose metabolism (Duran-Aniotz and Hetz, 2016).

Increased oxidative stress has been linked with decreased brain glucose uptake (Erukainure et al., 2019c). Increased glucose uptake has been shown to improve proteostasis which causes an upregulation of the unfolded protein response that protects against endoplasmic reticulum stress (Scheper and Hoozemans, 2015; Duran-Aniotz and Hetz, 2016). Oxidative stress has been implicated in the etiology and pathogenesis of neurodegenerative diseases (Erukainure et al., 2019b; Salau et al., 2020b). This is evident in the use of antioxidants in treating and managing most neurodegenerative diseases such as Alzheimer’s and Parkinson’s diseases (Gilgun-Sherki et al., 2001; Kim et al., 2015). Antioxidants have been reported for their ability to scavenge free radicals as well as improve the activities of the endogenous antioxidant enzymes (Salau et al., 2020). Oxidative stress has also been implicated in the disturbances of cholinergic and purinergic enzymes activities of the CNS (Erukainure et al., 2019a; Salau et al., 2020). These enzymes have been reported for their respective neurotransmission and bioenergetic roles which are critical for normal functioning of the brain (Ademiluyi et al., 2016; Pepeu and Giovannini, 2017).

*Cannabis sativa* L. is among the medicinal plants used in the treatment and management of neurological diseases. It is an annual herbaceous plant that belongs to the *Cannabis* genus and Cannabaceae family. It is globally distributed, with Africa accounting for 25% of its global production (UNODC, 2012). Its common names include weed, Indian hemp and marijuana. *C. sativa* is utilized for food, therapeutic, recreational and religious purposes (Bonini et al., 2018). Phytocannabinoids make up the major phytochemical constituents of *C. sativa*, with tetrahydrocannabinol (THC), cannabinol, and cannabidiol among the most common (Andre et al., 2016). The therapeutic role of *C. sativa* against neurodegenerative diseases and other psychopathic ailments have been reported (Lafuente et al., 2011; Campos et al., 2017; Lim et al., 2017). The aqueous and ethanol extracts of the leaves have been reported to confer protective effects on α-motor neurons via antioxidative and anti-apoptotic activities (Moosavi et al., 2013). Cannabidiol has been reported for its ability to activate metabotropic receptors for serotonin and/or adenosine as well as nuclear receptors of the PPAR family (Fernández-Ruiz et al., 2013). The leaves improved tremor, bradykinesia and rigidity in Parkinsonian patients (Lotan et al., 2014). The neuroprotective effects of *C. sativa* have been demonstrated on basal ganglia disorders (Sagredo et al., 2007). Other reported medicinal properties of *C. sativa* include anti-diabetes (Ren et al., 2016), anticancer (Guzman, 2003), pain suppression (Whiting et al., 2015), anti-epilepsy (Fusar-Poli et al., 2009), and sleep management (Ramar et al., 2018).

Despite the reported neuroprotective effect of *C. sativa* and its phytoconstituents, there are still dearth in its ability to promote brain glucose uptake and/or utilization. Thus, this present study was aimed at investigating the ability of the leaves to stimulate glucose uptake and utilization, as well as its modulatory effect on antioxidative, purinergic and cholinergic activities, and gluconeogenesis in isolated brains. The cytotoxic effect of the leaves was also investigated in glioblastoma multiforme (U87 MG) cells.

## Materials and Methods

### Plant Permit Approval

This research has been undertaken under the permit approval (Permit No. POS 248/2019/2020) from the South African Health Products Regulatory Authority to conduct, collect, posses, transport and store cannabis plant, plant parts and products for research purposes. The study was also conducted to collect cannabis plants in Lesotho under the permit (Permit #: 01/LS/2019/10/02-01).

### Plant Material

*Cannabis sativa* leaves were obtained from Mohale’s Hoek District, Lesotho (GPS coordinates: −30.333776″S and 27.651201″E). They were authenticated by the Geo Potts Herbarium at the University of the Free State, Bloemfontein 9300, South Africa and assigned the voucher number, BLFU MGM 0018. The leaves were pulverized to dry powder, after air drying to a constant weight.

The powdered samples were thereafter sequentially extracted with solvents of increasing polarity vis-à-vis hexane and dichloromethane (DCM) for 48 h with mild agitation of 100 rpm at room temperature. The solvents were respectively decanted and concentrated *in vacuo* using an R–215 rotary evaporator (Buchi, Switzerland). The extracts were collected in glass vials and stored in the dark at ambient room temperature for further *ex vivo* studies.

### Animals

A day to the experiment, sixteen male albino rats (Sprague Dawley strain) weighing 180 – 200 g were obtained from the Biomedical Research Unit, University of KwaZulu-Natal, Durban, South Africa and housed in plastic cages. They were fasted for 8 h before humanely sacrificed by euthanizing with Isofor. Their brains were harvested and rinsed in normal saline to remove blood stains. Each brain was divided into its hemispheres and used immediately for *ex vivo* study.

### Glucose Uptake in Isolated Rat Brain

Each hemisphere of the freshly harvested rat brains was incubated in 8 ml of Krebs buffer containing 11.1 mM glucose and the different concentrations of *C. sativa* extracts (hexane and DCM) for 2 h under a 5% CO_2_, 95% oxygen and 37°C conditions as described in previously published methods (Erukainure et al., 2019c; Salau et al., 2020a). The untreated control consisted of incubation without the extracts, while brains incubated without the extracts and glucose served as the normal control. Metformin was used as the standard drug. Each treatment group consisted of 3 brain hemispheres.

After incubation, the brain tissues were collected and homogenized in 50 mM phosphate buffer (pH 7.5) with 1% triton X-100, and thereafter centrifuged at 15,000 rpm at 4°C for 10 min (Erukainure et al., 2019c). The supernatants were collected and stored at −20°C until further analysis.

The study was carried out under the approved guidelines of the animal ethics committee of the University of KwaZulu-Natal, Durban, South Africa (protocol approval number: AREC/020/017D).

### Determination of Glucose Utilization

Aliquots from the incubating buffer was collected prior and after the incubation for determination of glucose utilization. This was carried out by measuring the glucose concentrations using an automated chemistry analyzer (Labmax Plenno, Labtest Inc., Lagoa Santa, Brazil). Glucose utilization was thereafter calculated with the formula:

$$\mathrm{Glucose uptake}=\frac{\mathrm{GC1}-\mathrm{GC2}\;}{\mathrm{Weight of brain tissue (g)}}$$

Where GC1 and GC2 are glucose concentrations (mg/dL) before and after incubation, respectively.

### Determination of Antioxidative Activity

#### Reduced glutathione level

The Ellman’s method (Ellman, 1959) was used in determining the GSH level of the brain tissue. Briefly, the resulting supernatant was deproteinized with an equal volume of 10% Trichloroacetic acid (TCA) and centrifuged for 5 min at 3,500 rpm. 200 μl aliquot was collected from the deproteinized sample into a 96 well plate. 50 μl of Ellman reagent was thereafter added and the reaction mixture was allowed to stand for 5 min. Absorbance was read at 415 nm. The GSH concentration was extrapolated from a standard curve.

#### Superoxide dismutase Activity

The superoxide dismutase (SOD) activity of the brain tissues was determined using a method based on the principle that 6-hydroxydopamine (6-HD) is oxidized by H_2_O_2_ from SOD catalyzed dismutation of O_2_^−^, which produces a colored product (Gee and Davison, 1989). Briefly, 15 μl of the tissue supernatants were dissolved in 170 μl of 0.1 mM diethylenetriaminepentaacetic acid (DETAPAC) in a 96 well plate. Thereafter, 15 μl of 1.6 mM 6-HD was added. Absorbance was measured at 492 nm for 5 min at 1 min interval.

#### Catalase Activity

The catalase activity of the brain tissue was determined using a previously established protocol (Aebi, 1984). Briefly, 10 μL of the tissue supernatants was mixed with 340 μL of 50 mM sodium phosphate buffer (pH 7.0). Thereafter, 150 μL of 2 M H_2_O_2_ was added to the reaction mixture. Absorbance was read at 240 nm at 1 min interval for 3 mins.

#### Lipid Peroxidation Level

The lipid peroxidation level of the brain tissues was determined by measuring the thiobarbituric acid reactive substances (TBARS) in the tissues and expressed as malondialdehyde (MDA) equivalent (Chowdhury and Soulsby, 2002). Briefly, a reaction mixture consisting of 100 μl of the supernatants, 100 μl of 8.1% SDS solution, 375 μl of 20% acetic acid, 1 ml of 0.25% thiobarbituric acid (TBA), and 425 μL of distilled water was heated at 95°C for 1 h in a water bath. A 200 μl aliquot was thereafter collected from the reaction mixture into a 96 well plate, and absorbance read at 532 nm.

### Nitric Oxide Level

The brain tissues were assayed for nitric oxide level using the Griess method as previously described (Tsikas, 2005; Erukainure et al., 2019c). Briefly, 100 μl of the tissue samples and/or distilled water (blank) were incubated with an equal volume of Griess reagent for 30 min at 25°C in the dark. Absorbance was read at 548 nm.

### Cholinergic Enzymes Activities

The brain tissues were assayed for cholinergic activities by analyzing the activities of acetylcholinesterase (Ellman et al., 1961) and butyrylcholinesterase (Adefegha et al., 2017) respectively in the tissue supernatants. Briefly, 20 μl of the tissue supernatants was incubated with 10 μl of 3.3 mM Ellman’s reagent (pH 7.0) and 50 μl of 0.1 M phosphate buffer (pH 8) for 20 min at 25°C. For acetylcholinesterase activity, the reaction was stopped by adding 10 μl of 0.05 M acetylcholine iodide to the reaction mixture. While 10 μl of 0.05 M butyrylcholine iodide was used to stop the reaction for butyrylcholinesterase activity. Absorbances were read at 412 nm at 3 min intervals.

### Purinergic Enzymes Activities

Th brain tissues were assayed for purinergic activities by analyzing for the activity of ecto-nucleoside triphosphate diphosphohydrolase (E-NTPDase) (Akomolafe et al., 2017) in the tissue supernatants. Briefly, 20 µl of the tissue supernatants was incubated with 200 µl of the reaction buffer (1.5 mM CaCl_2_, 5 mM KCl, 0.1 mM EDTA, 10 mM glucose, 225 mM sucrose and 45 mM Tris-HCl) at 37°C for 10 min 20 µl of 50 mM ATP was added to the reaction mixture and further incubated at 37°C for 20 min in a shaker. The reaction was stopped with 200 µl of 10% TCA and incubated on ice for 10 min. Absorbance was read 600 nm.

### Determination of Gluconeogenic Enzymes Activities

The gluconeogenic enzymes activities of the brain tissues were determined by analyzing the supernatants for glucose 6 phosphatase (Mahato et al., 2011; Erukainure et al., 2017), and fructose-1,6-bisphosphatase (Gancedo and Gancedo, 1971; Balogun and Ashafa, 2017) activities with slight modifications.

To determine glucose 6 phosphatase activity, 200 µl of the tissue supernatants was incubated with 100 µl of 0.25 M glucose, 200 µl of 5 mM KCl, 1300 µl of 0.1 M Tris-HCl buffer, and 40 µl of 50 mM ATP at 37°C in a shaker for 30 min. The reaction was stopped with 1 ml of distilled water and 1.25% ammonium molybdate. 1 ml of freshly prepared 9% ascorbic acid was then added to the reaction mixture and allowed to stand for 30 min. Absorbance was read at 660 nm. ATPase activity was calculated as the amount of inorganic phosphate (Pi) released/min/mg protein.

For fructose-1,6-biphosphatase activity, 100 µl of the tissue supernatant was incubated with 1,200 μl of Tris–HCl buffer (0.1 M, pH 7.0), 100 μl of fructose (0.05 M), 250 μl 0.1 M MgCl_2_, 100 μl 0.1 M KCl, and 250 μl 1 mM EDTA at 37°C for 15 min. The reaction was stopped with 1 ml of 10% TCA and incubated on ice for 10 min. Absorbance was read at 680 nm and the activity calculated as the amount of inorganic phosphate (Pi) released/min/mg protein.

### 3-(4,5-Dimethylthiazol-2-yl)-2,5-Diphenyltetrazolium Bromide Assay

#### Cell Lines and Cell Culturing

The human U87 MG glioblastoma cancer (ATCC^®^ HTB-14™) cell line was obtained from the American Type Culture Collection (ATCC, Virginia, United States). Dulbecco's Minimum Essential Media (DMEM), Eagle’s Minimum Essential Media (EMEM), Fetal bovine serum (FBS) and Phosphate Buffer Saline (PBS) were purchased from Life technologies (Pty) Ltd. (Fairlands, Johannesburg, RSA). The 3-(4,5-Dimethylthiazol-2-yl)-2,5-Diphenyltetrazolium Bromide (MTT), Dimethyl Sulfoxide (DMSO), Trypsin, and all other chemicals and reagents were of analytical grade and acquired from Merck (Pty) Ltd. (Modderfontein, Johannesburg, RSA). The U87 cell line was maintained in DMEM:EMEM (1:1), supplemented with 10% heat-inactivated FBS and grown at 37°C in a humidified incubator set at 5% CO2. Cells were sub-cultured with 0.25% (w/v) trypsin 0.53 mM ethylenediaminetetraacetic acid (EDTA) for a maximum of 15 min every 2 and 3 days after they had formed an 80% confluent monolayer.

#### Cell Proliferation Assay

To analyze the effect of the samples on the cell viability, the MTT method was utilized. Cells were seeded in 100 µl medium in 96-well microtiter plates at a concentration of 1 × 10^5^. Stock solutions of 4 mg/ml of the samples were prepared in 20% ethanol. The positive control, doxorubicin was dissolved in 50% DMSO to obtain a stock concentration of 10 mg/mL. Serial dilutions were made to achieve target concentrations of the samples and doxorubicin in a of range 30, 60, 120, 240 μg/ml and 12.5–100 μg/ml, respectively. Subsequently, cells were exposed to the samples, doxorubicin and the controls, which included vehicle-treated cells exposed to 1.2% ethanol or 0.5% DMSO and cells propagated in growth medium. After the 48 h treatment period, the cells were subjected to the МТТ reagent (0.5 mg/mL). The colorimetric reaction was measured by means of a plate reader (Multiskan Go, Thermofischer Scientific) at 570 nm wavelength. Color control blanks were included and utilized to normalize the results and the vehicle control treated cells were regarded as 100% cell viability. The samples were evaluated in atleast three independent experimental repeats and each sample was evaluated in triplicate for each experimental repeat. The results given are representative of the average percentage inhibition of all the experimental repeats.

### GC-MS analysis of *Cannabis sativa* Extracts

#### Apparatus

An Agilent technologies 6890N GC-MS machine coupled with a 5973-network mass selective detector was used for the analysis. An Agilent technologies 7683 Series injector and screw neck glass vials (separations) were used for sample injection.

#### GC-MS Conditions

The extracts were analyzed using a HP-5ms capillary column phase. The injection volume was set to 2 µl at 80°C. the flow rate of the helium gas was 1 ml/min at a constant flow. The oven program was set at 80°C for 2 min and increased to 300°C at 20°C/min for 3 min. A mass selective detector was used for the detection of the volatile compounds present in the standards and tests samples. The NIST mass spectral library database software was used to analyze the data. The mass spectrometer data system has a reporting software module that combines the data results with the NIST library and ADMIS software search for target compounds into a single report.

#### *In Silico* Prediction of Blood Brain Barrier Permeability and Oral Lethal Dose Toxicity

The ability of the GC-MS identified compound to cross the BBB was predicted *in silico* using the SwissADME online server (http://www.swissadme.ch/index.php) (Daina et al., 2017). Its oral lethal dose toxicity (LD_50_) and toxicity class were predicted using the PROTOX II online server (http://tox.charite.de/protox_II/) (Banerjee et al., 2018). These were done by obtaining the Canonical SMILES of the GC-MS identified compound from PubChem (https://pubchem.ncbi.nlm.nih.gov/), and computed into the respective servers for the predictions.

### Statistical Analysis

Data were subjected to one-way ANOVA and presented as mean ± SD. Significant differences between means at *p* < 0.05 were obtained using the Tukey’s HSD-multiple range post-hoc test. Statistical analyses were done using IBM Statistical Package for the Social Sciences (SPSS) for Windows, version 23.0 (IBM Corp., Armonk, NY, United States).

## Results

**FIGURE 1:**
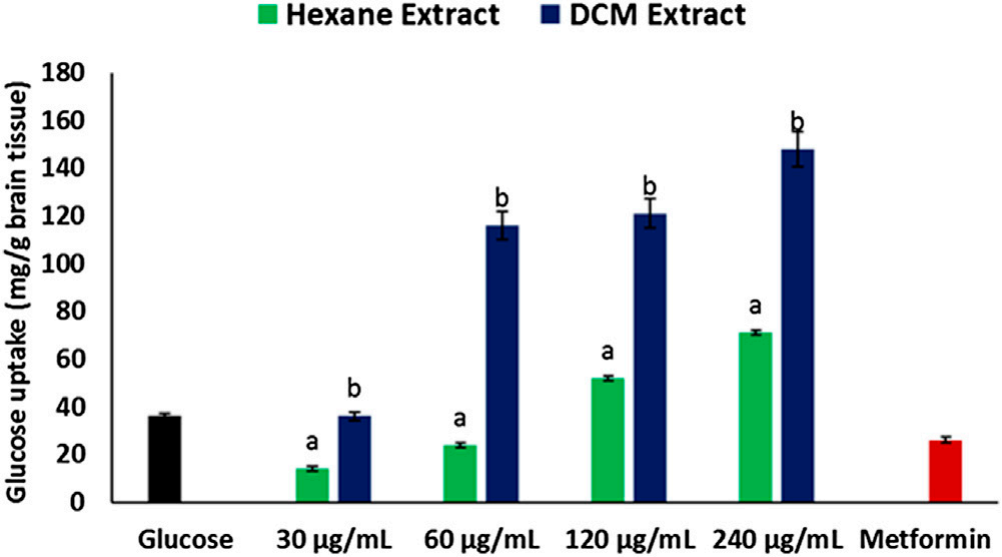
Effect of *Cannabis sativa* on glucose uptake in brain tissues. *^ab^Values with different letter above the bars for a given extract are significantly (*p* < 0.05) different from each other.

Incubation of brain tissues with glucose in the presence of hexane and DCM extracts of *C. sativa* leaves led to significant (*p* < 0.05) increase in glucose utilization, with the DCM extract having a higher activity as depicted in Figure 1. The activity was dose-dependent, with the highest concentration having the highest activity. Incubation with metformin had no significant effect on brain glucose utilization.

**FIGURE 2:**
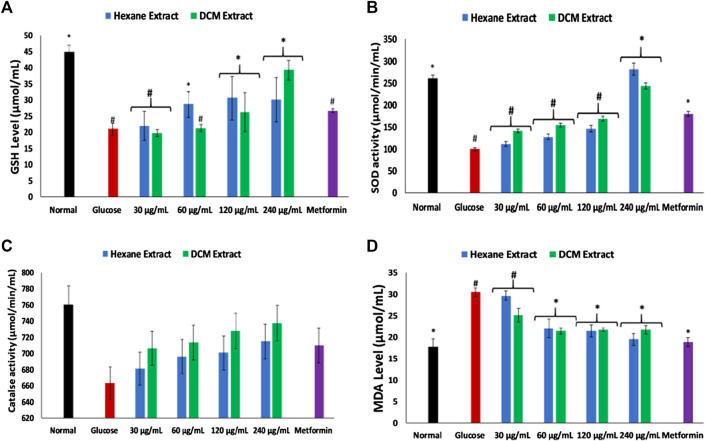
Effect of *Cannabis sativa* on **(A)** glutathione (GSH) level, **(B)** superoxide dismutase (SOD) activity, **(C)** catalase activity, and **(D)** malondialdehyde (MDA) level in brain glucose uptake. Value = mean ± SD; n = 3. *Statistically significant (*p* < 0.05) compared to glucose-treated tissue; #statistically significant (*p* < 0.05) compared to normal tissue. Normal, No glucose/*C. sativa*.

Incubation of brain tissues with glucose led to significant (*p* < 0.05) depletion in the levels of GSH, SOD and catalase activities, while significantly elevating MDA level as shown in Figures 2A–D. Incubation with the extracts significantly (*p* < 0.05) reversed these levels and activities dose-dependently to near normal.

**FIGURE 3:**
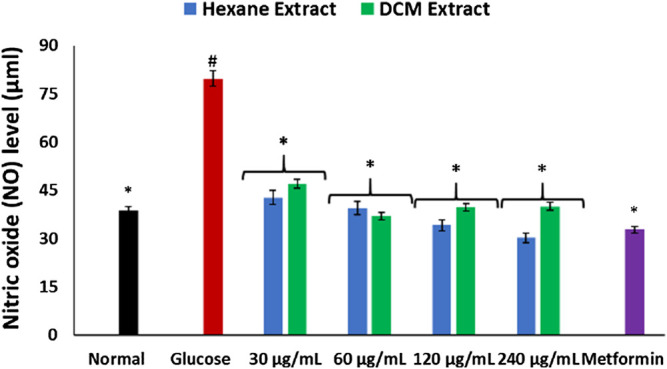
Effect of *Cannabis sativa* on NO level in brain glucose uptake. Values = mean ± SD; n = 3. *Statistically significant (*p* < 0.05) compared to glucose-treated tissue; #statistically significant (*p* < 0.05) compared to normal tissue. Normal, No glucose/*C. sativa*.

As shown in Figure 3, incubation with glucose led to significant (*p* < 0.05) elevation of NO level in brain tissues. This level was significantly (*p* < 0.05) depleted on incubation with *C. sativa* extracts.

**FIGURE 4:**
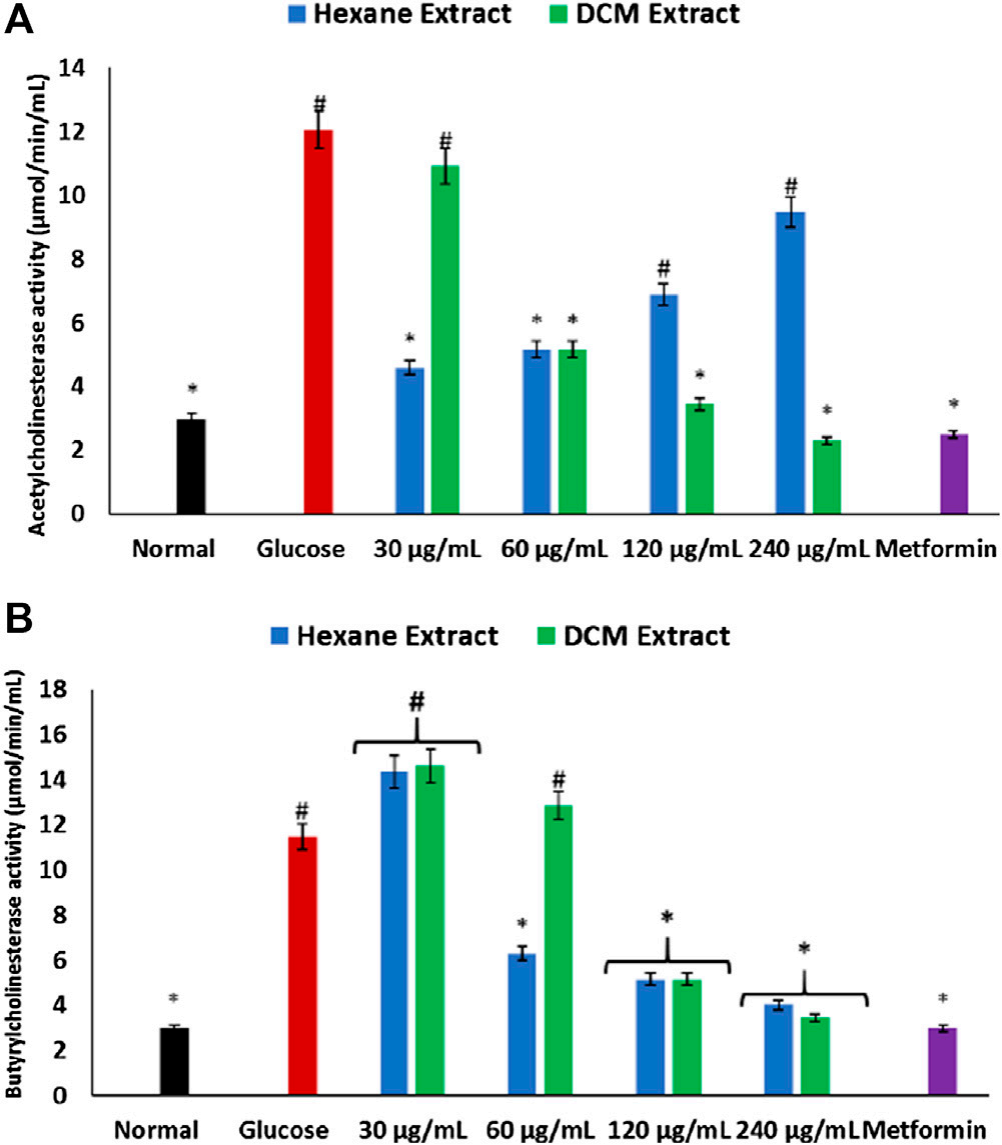
Effect of *Cannabis sativa* on **(A)** acetylcholinesterase and **(B)** butyrylcholinesterase activities in brain glucose uptake. Values = mean ± SD; n = 3. *Statistically significant (*p* < 0.05) compared to glucose-treated tissue; #statistically significant (*p* < 0.05) compared to normal tissue. Normal, No glucose/*C. sativa*.

There was a significant (*p* < 0.05) elevation in acetylcholinesterase and butyrylcholinesterase activities in brain tissues incubated with glucose as depicted in Figures. 4A,B. The acetylcholinesterase activity was significantly (*p* < 0.05) reversed dose-dependently on incubation with DCM extract (Figure 4A). However, the hexane extract significantly (*p* < 0.05) elevated acetylcholinesterase activity dose-dependently (Figure 4A). Both extracts significantly (*p* < 0.05) depleted butyrylcholinesterase activity in a dose-dependent manner (Figure 4B).

**FIGURE 5:**
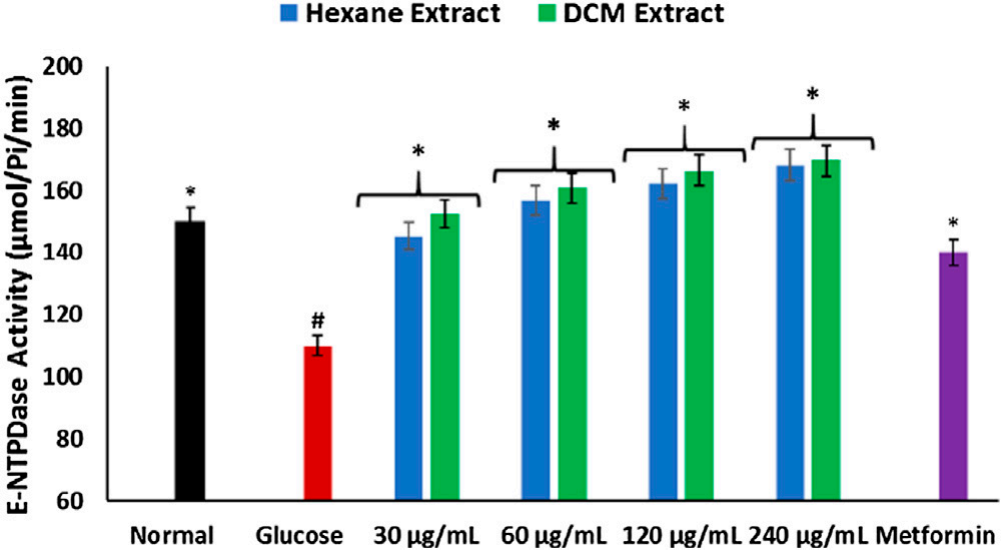
Effect of *Cannabis sativa* on ecto-nucleoside triphosphate diphosphohydrolase activity in brain glucose uptake. Values = mean ± SD; n = 3. *Statistically significant (*p* < 0.05) compared to glucose-treated tissue; #statistically significant (*p* < 0.05) compared to normal tissue. Normal, No glucose/*C. sativa*.

As shown in Figure 5, there was a significant (*p* < 0.05) depletion in E-NTPDase activity in brain tissues incubation with glucose only. Incubation with *C. sativa* extracts led to significant (*p* < 0.05) reversion of the activity to levels indistinguishable from the normal tissues.

**FIGURE 6:**
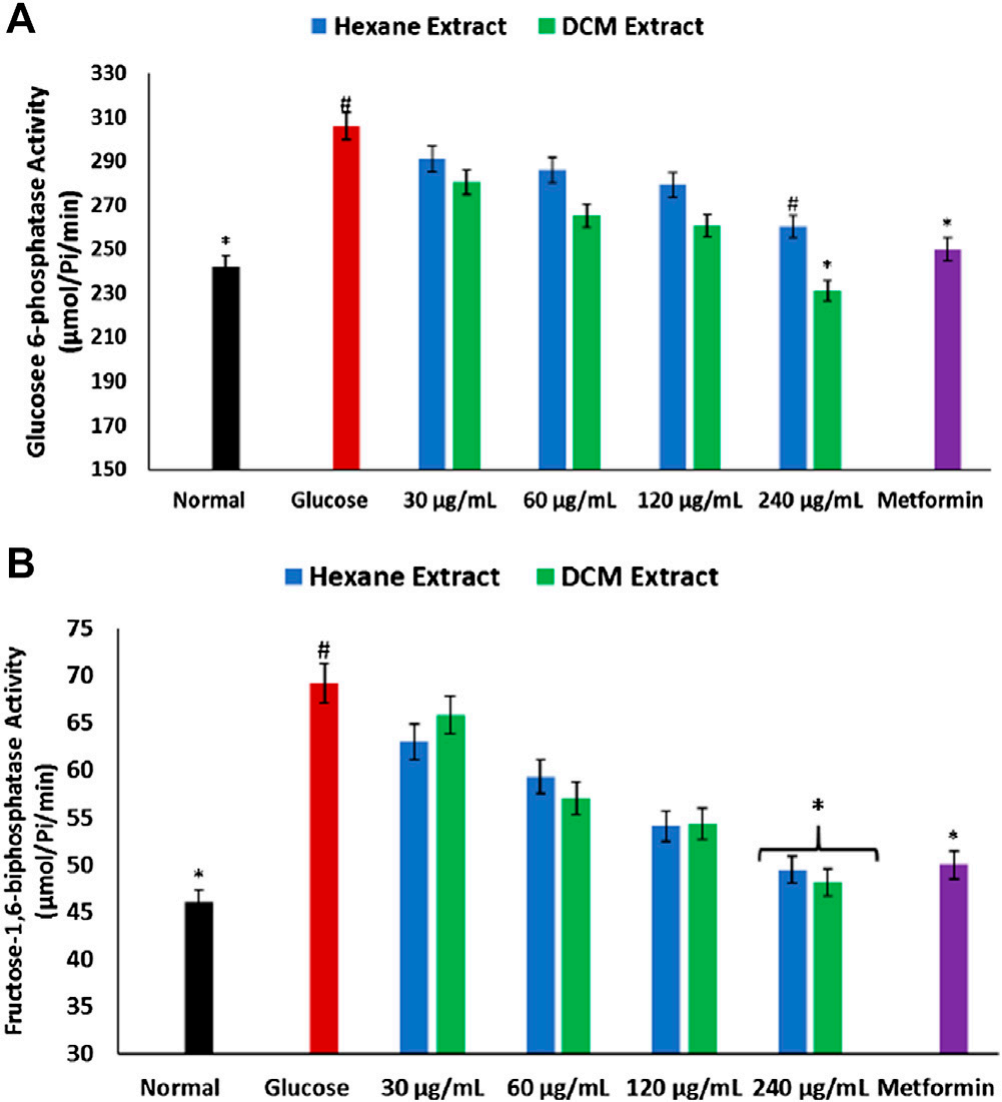
Effect of *Cannabis sativa* on **(A)** glucose 6-phosphatase and **(B)** fructose-1,6-biphosphatase activities in brain glucose uptake. Values = mean ± SD; n = 3. *Statistically significant (*p* < 0.05) compared to glucose-treated tissue; #statistically significant (*p* < 0.05) compared to normal tissue. Normal, No glucose/*C. sativa*.

As depicted in Figures. 6A,B, there was a significant (*p* < 0.05) elevation in the activities of glucose 6-phosphatase and fructose-1,6-biphostase in brain tissues incubated with glucose only. These activities were significantly (*p* < 0.05) reversed in tissues incubated with *C. sativa* extracts to levels indistinguishable from the normal tissues.

**FIGURE 7:**
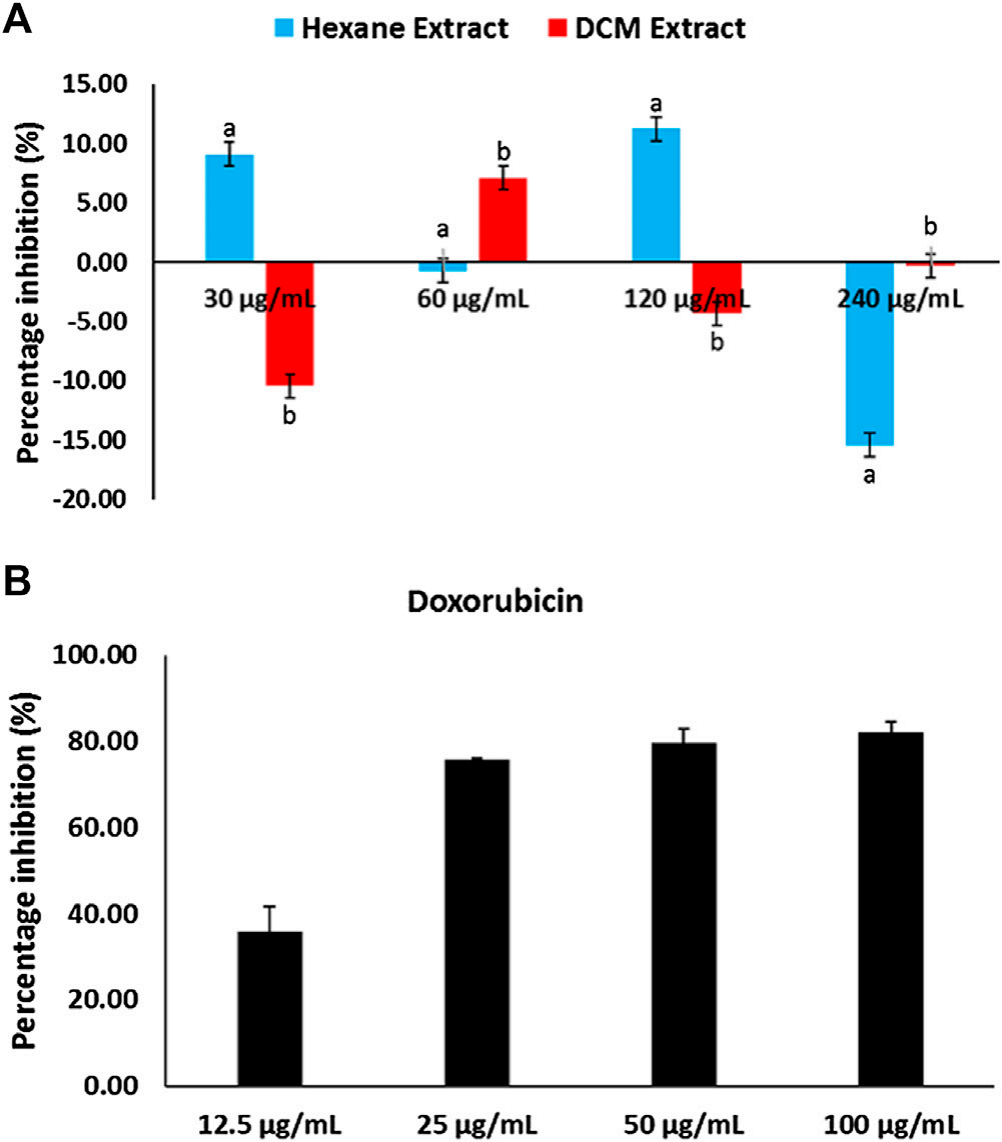
Cytotoxic effect of **(A)** *Cannabis sativa* and **(B)** doxorubicin on glioblastoma multiforme (U87 MG) cells. Values = mean ± SD; n = 3.

MTT assay revealed *C. sativa* extracts had little or no cytotoxic effect on U87 MG cells, while doxorubicin significantly (*p* < 0.05) inhibited the proliferation of the cells as depicted in Figure 7.

**FIGURE 8:**
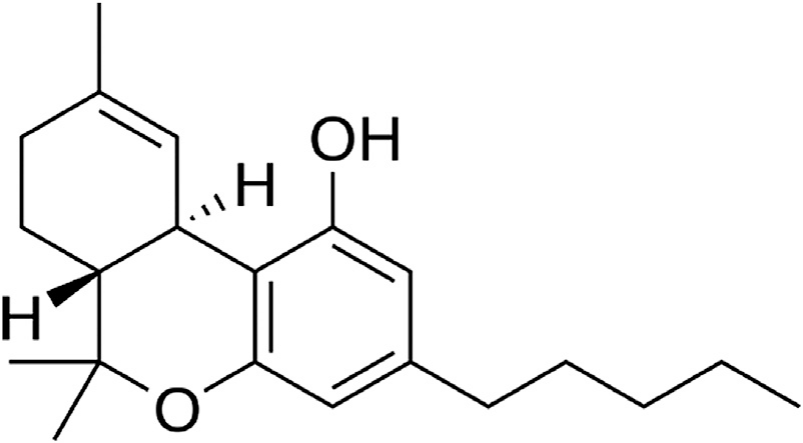
Tetrahydrocannabinol identified from the hexane and DCM extracts of *C. sativa*.

GC-MS analysis of the extracts revealed the presence of THC in both extracts as shown in Figure 8; Supplementary Figure S1.

*In silico* BBB permeability prediction revealed THC to be permeable across the BBB as shown in Table 1. THC was further predicted *in silico* to be in the toxic class of 4, with a LD_50_ value of 482 mg/kg.

**TABLE 1:** **** In silico prediction of BBB permeability and toxicity of tetrahydrocannabinol.

*In Silico* activity	Predictions
LD_50_	482 mg/kg
Toxicity class	4
BBB permeability	Yes

BBB, blood brain barrier; LD_50_, ethal dose.

## Discussion

Impaired brain glucose uptake leading to diminished neuronal glucose consumption has been linked to the pathogenesis and progression of neurodegenerative diseases such as Alzheimer’s and Parkinson’s diseases (Zilberter and Zilberter, 2017; An et al., 2018). In the present study, the increased glucose uptake in brain tissues incubated with *C. sativa* extracts (Figure 1) indicates an increased glucose utilization which insinuates an increased neuronal glucose consumption. This activity portrays a facilitative uptake potential of *C. sativa* leaves which corresponds with previous studies on the use of medicinal plant leaves in facilitating brain glucose uptake (Erukainure et al., 2019c). This activity can be attributed to the GC-MS identified compound in the extracts, THC (Figs. 8 and S1) which can bind and activate the cannabinoid receptors of the endocannabinoid anandamide transporters at the BBB (Maccarrone et al., 2006). Thus, facilitating transportation across the BBB.

Oxidative stress and proinflammation have been implicated in the pathogenesis of neurological diseases, and have been reported among the major mechanisms in the etiology of the diseases (Wajner et al., 2004; Mahadik et al., 2006). The depleted GSH level, SOD and catalase activities on incubation of brain tissues with glucose (Figures. 2A–C) insinuates an oxidative state. This is further revealed by the exacerbated MDA level (Figure 2D) which indicates an occurrence of lipid peroxidation. This is in agreement with previous studies on exacerbated oxidative activities in brain tissues incubated with glucose (Erukainure et al., 2019c). The brain has been reported for its high susceptibility to oxidative stress owing to its polyunsaturated fatty acids contents, redox-active metal load, low endogenous antioxidant system, and auto-oxidizable neurotransmitters dependence (Butterfield et al., 2001; Huang et al., 2004; Patel, 2016; Erukainure et al., 2019c). The increased brain NO level (Figure 3) with concomitant low SOD activity on incubation with glucose may insinuate a proinflammatory effect. In the presence of depleted SOD activity, peroxynitrite (ONOO−) is generated from the reaction of NO and superoxide (O2−) (Erukainure et al., 2020; Salau et al., 2020). Peroxynitrite has been reported for its potent proinflammatory roles in several diseases including neuropathy as it has been implicated in the pathogenesis of Alzheimer’s and Parkinson’s diseases, and multiple sclerosis (Smith et al., 1997; Pacher et al., 2007). Antioxidants have been reported for their therapeutic roles in the treatment and management of neurodegenerative diseases (Gilgun-Sherki et al., 2001; Kim et al., 2015). The elevated GSH level, SOD and catalase activities, with concomitant depleted levels of MDA and NO on incubation with *C. sativa* extracts indicate an antioxidative and anti-proinflammatory effect. THC has been reported for its potent antioxidant and anti-proinflammatory activities in the treatment and management of neurological diseases (Hampson et al., 1998; Costa, 2007; Borges et al., 2013). Thus, may be responsible for the antioxidative effect of the extracts.

Cholinergic dysfunction has been recognized as one of the major defects of neurodegenerative diseases such as Alzheimer’s, Parkinson’s diseases and multiple sclerosis (Greig et al., 2002). It is characterized by increased activities of acetylcholinesterase and butyrylcholinesterase which catalyze the hydrolysis of the neurotransmitter acetylcholine (Reid et al., 2013). Thus, implying that the elevated activities of these enzymes on incubation of brain tissues with glucose (Figures 4A,B) indicates a cholinergic dysfunction and may insinuate a neurodegenerative symptom. Several therapies have targeted the inhibition of these enzymes in the treatment and management of neurodegenerative diseases (Erukainure et al., 2019a; Salau et al., 2020b). Thus, the inhibitory effect of the extracts on these enzymes portrays a neuroprotective activity of *C. sativa*. This corroborates previous reports on the inhibitory effect of *C. sativa* and its major phytochemical constituents on acetylcholinesterase and butyrylcholinesterase activities (Eubanks et al., 2006; Abdel-Salam et al., 2018). However, the increasing acetylcholinesterase activity with increasing concentration of the hexane extract (Figure 4A) may insinuate the inhibitory effect of the extract on the enzyme diminishes with increasing concentrations.

The depleted E-NTPDase activity in brain tissues incubated with glucose (Figure 5) indicates a depleted adenosine level which portrays a decreased purinergic activity. Impaired purinergic activity has been implicated in the pathogenesis of neurodegenerative diseases (Akomolafe et al., 2017; Salau et al., 2020). It is characterized by decreased production of adenosines which are involved in energy transfer reactions and facilitative transportation (Akomolafe et al., 2017). The increased E-NTPDase activity in brain tissues incubated with *C. sativa* extracts insinuates the ability of *C. sativa* to improve neuronal purinergic activity.

Diminished brain glucose consumption is often characterized by increased glycogenolysis and impaired glycolytic flux to compensate for the low glucose level (Hoyer, 1996; Atlante et al., 2017). This has been implicated in the pathogenesis of Alzheimer and other neurodegenerative diseases (Atlante et al., 2017). In the present study, the depleted brain glucose consumption (Figure 1) corroborates with the exacerbated glucose 6-phosphatase and fructose-1,6-biphosphatase activities in brain tissues incubated in glucose (Figures. 6A,B). Both enzymes are involved in gluconeogenesis, with glucose 6-phosphatase and fructose-1,6-biphosphatase catalyzing the hydrolysis of glucose 6-phosphate to glucose in the glycogenolytic pathway and fructose-1,6-biphosphate to fructose 6-phosphate in the gluconeogenic pathway respectively. The continuous activation of these enzymes will lead to glucose accumulation which can serve as metabolite precursors for the hexosamine, polyol, protein kinase C, and AGE pathways which have been linked to the pathogenesis of neurodegenerative diseases (Li et al., 2012; Xu et al., 2016). Glucose has also been reported as an intermediate for the generation of free radicals, as it is oxidized in its enediol form into reactive ketoaldehydes and superoxide anion radicals (Maritim et al., 2003). Thus, the dose-dependent inhibited activity of these enzymes in brain tissues incubated with *C. sativa* extracts further indicates the neuroprotective effect of *C. sativa.*

Glioblastoma multiforme (GBM) is a malignant primary brain tumor common in young kids (Erukainure et al., 2018). Studies have reported therapeutic failures in patients owing to difficulty in treatment (Puli et al., 2006). Although medicinal plants have reported to arrest the proliferation of GBM (Erukainure et al., 2018), *C. sativa* extracts showed no cytotoxic effect on the cells (Figure 7). Thus, insinuating that the plant may not be beneficial in the treatment of GBM.

The predicted ability of THC to permeate the BBB (Table 1) further connotes the neuroprotective effect of *C. sativa* and corroborates previous reports on the ability of cannabinoids to modulate the cannabinoid receptors of anandamide transport across the BBB (Maccarrone et al., 2006). The predicted oral LD_50_ and toxicity class values of THC (Table 1) indicates that the compound is relatively safe when orally consumed.

## Conclusion

As portrayed by these results, *C. sativa* improves glucose consumption with concomitant suppression of oxidative stress and cholinergic dysfunction, and modulation of purinergic and gluconeogenic activities in brain tissues. Further studies are recommended to decipher the molecular mechanisms that may be involved in these neuroprotective activities in *in vivo* studies.

## Data Availability Statement

The original contributions presented in the study are included in the article/Supplementary Material, further inquiries can be directed to the corresponding author.

## Ethics Statement

The animal study was reviewed and approved by The animal ethics committee of the University of KwaZulu-Natal, Durban, South Africa (protocol approval number: AREC/020/017D).

## Author Contributions

MM and OE conceptualized and designed the research project; OE, VS and MI carried out the experiments; MM and OE wrote the original manuscript; all authors revised and approved the final manuscript draft; MM supervised the project.

## Funding

Authors are thankful to IKS Based Technology Innovation Unit of DSI South Africa, for financial support (Grant contracts: DST/CON 0162/201 and DST/CON 0206/2019/2020) and University of the Free State for technical support and postdoctoral support for OE.

## Conflict of Interest

The authors declare that the research was conducted in the absence of any commercial or financial relationships that could be construed as a potential conflict of interest.
