# Supplementary material for: Tetrahydrocannabinol-Rich Extracts From Cannabis Sativa L. Improve Glucose Consumption and Modulate Metabolic Complications Linked to Neurodegenerative Diseases in Isolated Rat Brains
Source: Front Pharmacol. 2020 Nov 24;11:592981. doi: 10.3389/fphar.2020.592981 (PMC7774498; doi:10.3389/fphar.2020.592981)
Supplement: Supplementary file 1 [file datasheet1.pdf]

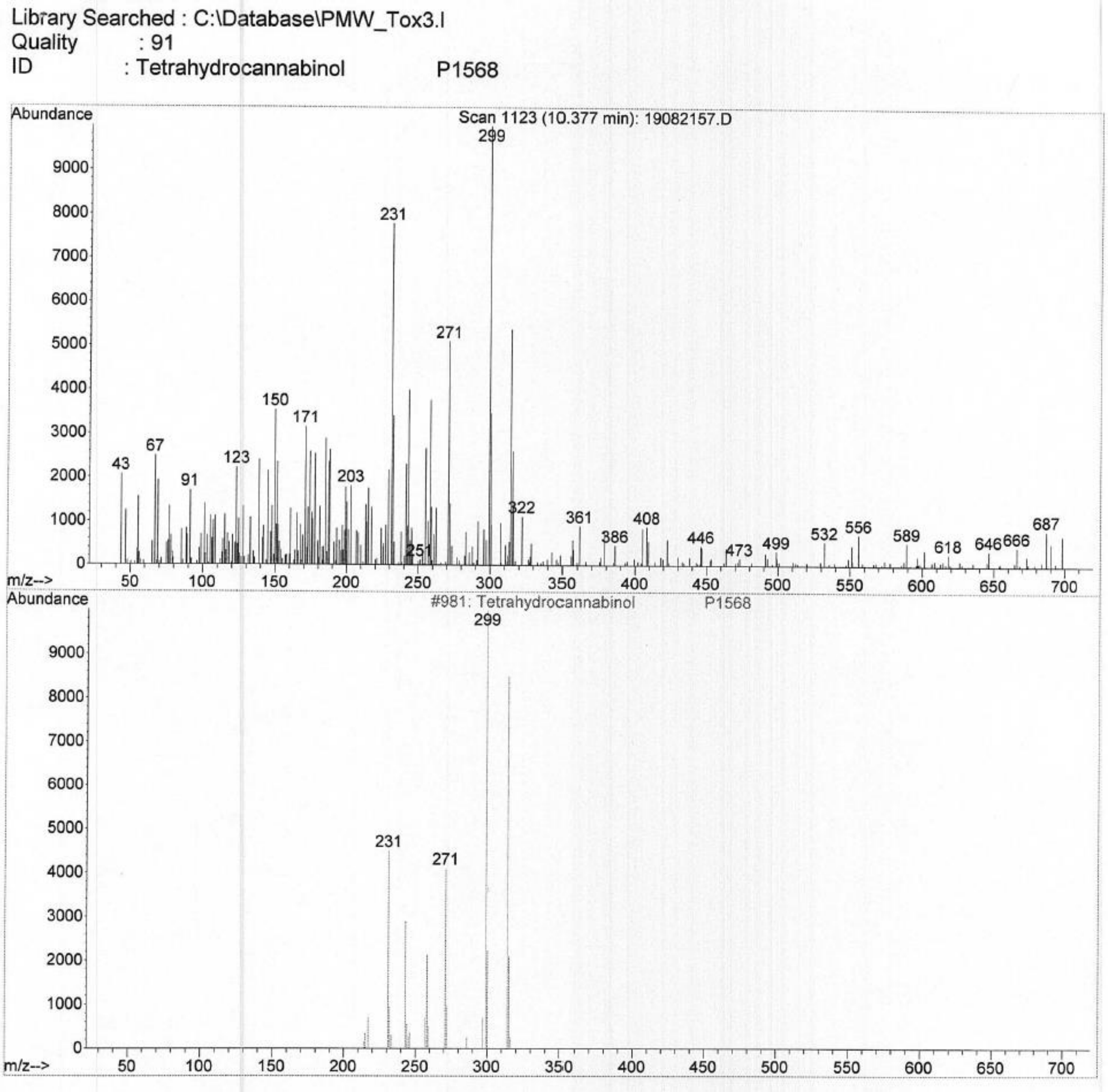

Fig. S1A: GC-MS chromatogram of *C. sativa* hexane extract

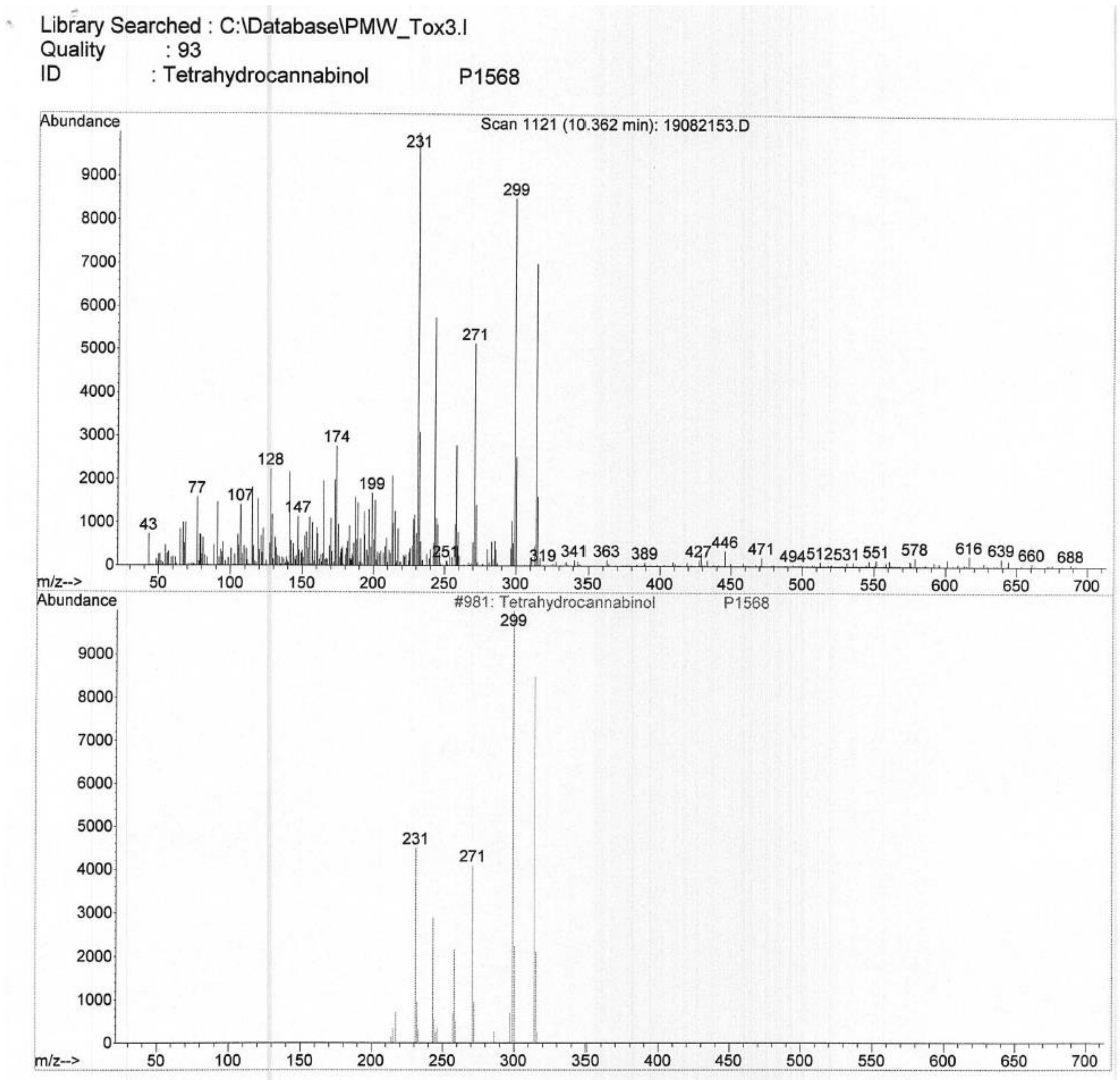

Fig. S1B: GC-MS chromatogram of *C. sativa* DCM extract
